# Paradoxes of indigeneity: identity, the state, and the economy in Indonesia

**DOI:** 10.1007/s10624-021-09640-7

**Published:** 2021-11-30

**Authors:** Timo Duile

**Affiliations:** Department of Southeast Asian Studies, Bonn University, Bonn, Germany

**Keywords:** Indigeneity, Indigenous paradox, South Sulawesi, Indonesia, Political economy

## Abstract

Contributing to recent debates on indigeneity, this article investigates contradictions of indigeneity, especially the “indigenous paradox,” that is, the formation of indigeneity through claiming sovereignty and autonomy from the state by acknowledging the very state and its laws as the framework for those claims, in the context of Indonesia. After analyzing how indigeneity came into existence in the Indonesian context, this article sheds light on the process of indigenous recognition in the Duri highlands, South Sulawesi. It is argued that the contradictions of indigeneity concern not only indigenous—state relations, but also narratives on tradition and history, and most of all, economic contradictions. It is the recognition of the overall framework of capitalism and the state which makes possible the emergence of alternative local economies based upon solidarity. Drawing on Louis Althusser’s concept of overdetermination, this article suggests that indigeneity shapes the way how economic contradictions are expressed, and while it provides local spaces for alternative economies, indigeneity is also prone to being incorporated into the logics of capitalism.

## Introduction

The phenomenon of indigeneity has often been described as a relational identity which appears as a given but actually emerges in relation to other, mostly oppressive, forces such as the state or settler societies. Recently, Jonas Bens (2020) argued that native communities become indigenous when they occupy a paradoxical legal position. Referring to cases in the Americas, Bens explains that on the one hand, indigenous peoples reject the state and view the regulation of their affairs by the state as a violation of their sovereignty and autonomy. This rejection is not a universal state of affairs, but in many cases, indigenous organizations emphasize at least some kind of opposition to the postcolonial state as it often restricts indigenous people’s sovereignty. On the other hand, indigenous communities usually depend on the state for their existence as they need recognition from the state and therefore have to accept and recognize the state as the ultimate legal framework within which they can make claims (Bens 2020: 2–3). This contribution outlines paradoxical legal formation in the context of Indonesia and argues that other dimensions of the paradoxical structure of indigeneity have to be taken into account. Beside the notion of becoming indigenous *with and against* the state, there is a historical/narrative dimension of the indigenous paradox. This dimension describes how indigeneity has come into existence through history and narratives on history. On the one hand, indigeneity emerges as an original identity, but it is also clear that in the notion of relatively egalitarian, small communities bound to a certain place and marginalized from mainstream society came into existence only through a history of rapid and violent change, and indigeneity has incorporated this change in order to appear as original: What appears as indigenous now is also the result of anti-traditional forces. This dimension of the indigenous paradox describes indigeneity as an identity coming into being *with and against* hegemonic narratives of history. Second, and (in my view) more importantly, there is an economic dimension of the indigenous paradox which emerges when indigeneity is expressed in terms of an alternative economy. The foremost aim of indigenous recognition is, in many places, the recognition of indigenous land rights (which are a main means of production). Land controlled by the state should be transferred to indigenous communities. But it is not always clear how this land is then managed, and indigenous NGOs and communities have engaged in debates over what makes an (local) economy “indigenous.” These ideas of indigenous economies are potentially paradoxical as well: they reject state control and capitalism, but at the same time, alternative forms of local economies of solidarity can only emerge if state control and capitalism are accepted as the very framework for them. As a result, concepts of alternative local economies often remain vague, and many activists use images of the indigenous entrepreneur in order to gain support from local state apparatuses. But within that framework economies of communal ownership of means of production and self-administration become possible. It is this acceptance of the state and its ideology (of economic growth and entrepreneurship, for instance) that make it possible for alternative local economies to emerge.

This article outlines these dimensions of the indigenous paradox in regard to Indonesia, the history of indigeneity in this country, and sheds light especially on the process of becoming indigenous in the Duri highlands in South Sulawesi. In order to understand the economic foundation of the indigenous paradox, it draws on the Althusserian concept of overdetermination (Althusser 2005). Economic contradictions and conditions do not solely determine identities and political process. Rather, economic factors are determinants in the last instance, but never occur in their pure forms. This is a useful argument when analyzing indigeneity and indigenous rights from a critical perspective, taking into account the political economy into which they are embedded (Goodale 2016).

This article is based on findings from field work during the author’s stay as a guest researcher at Hasanuddin University in Makassar between January and December 2019, during which research on the indigenous movement in Jakarta, Makassar, and Enrekang was carried out. This was complemented by online conversations with interlocutors during the coronavirus pandemic. It outlines overall trends on the national scale and analyzes how they shape concrete processes of becoming indigenous at the grassroots level.

## The indigenous paradox: contradictions and overdetermination

As this article aims to contribute to ongoing debates on indigeneity, it departs from some current analysis of indigenous identity. In order to come into existence as a political force, indigenous activists often understand indigeneity as simply a matter of descent and kinship, and this descent establishes a relation to the land they claim (cf. Bens 2020: 4). In these views, indigeneity is a matter of identity as fixed and given entities. In contrast, some scholars have stressed that indigeneity comes into existence through and in relation to autochthon communities and settler societies or postcolonial states, and some indigenous scholars have adopted such approaches as well (for instance Mikdasih 2013; Simpson 2017; Corntassel 2018). In these accounts, indigeneity emerges as what Merlan (2007) has labeled a relational identity. Indigeneity always needs its other (the state, the setter colony, or mainstream society). In all these cases, an oppressive relationship had been established which marginalized people and communities economically, politically, and culturally, and indigeneity is the result of these processes. However, scholars critical of indigenous movements have stressed that indigeneity is not an identity in and of itself. In these analyses, indigeneity emerges as a form of ethnonationalism against other parts of the population (most prominently: Kuper 2003a, 2003b). While this is not necessarily or always the case (and the example of AMAN in Indonesia proves that indigeneity does not necessarily rely on ethnonationalism), indigeneity, when conceptualized as an ethnic identity with a notion of being original to a place, holds the potential to draw boundaries of identity similar to those of European ethnonationalism where language, origin, or ethnicity are essentialized.

Taking these approaches into account, Jonas Bens has analyzed what he calls the “indigenous paradox.” In his work on indigenous court cases in the Americas, he concludes that indigeneity inevitably presents itself as a paradox. In order to emerge as indigenous, a community usually has to claim independence from (or sovereignty against) the state in the sense of being distinct in cultural terms and/or has to apply the extralegal fiction of cultural difference (Bens 2020: 190–191). In Bens’ account, the paradoxical formation of indigeneity emerges as a matter of law: indigenous communities claim sovereignty from the law of the nation states they find themselves in and apply this very law in order to make their claims. The relational character of indigeneity is, in this regard, a matter of rights and sovereignty. As such, it is a juridical paradox. The indigenous paradox is based upon a contradiction between being part of the state and law and claiming a position outside it.

Indigeneity and its paradoxes can be interpreted either in a poststructuralist or in a dialectical way. What I call the post-structuralist way here are those approaches that are concerned with pure difference and this difference usually emerges through relations (which can be oppositions but also, for instance, chains of equivalences that are related to oppositional chains of equivalences). The main idea in these approaches is that there is nothing but difference and relations, and thus meaning and identity are only possible through this difference. This is an approach of radical difference. Dialectics works differently: in a dialectical approach, identities are also not self-identical, but in contrast to relational approaches, a dialectical approach stresses that difference is always part of what it opposes. It is an approach of relative difference. In other words, identities include self-difference. Much of the recent work I have mentioned above stresses the relational character of indigeneity and as such it is (explicitly or implicitly) influenced by poststructuralist approaches. Indigeneity is difference and the difference of indigeneity as a signifier exists through its relation to other signifiers, such as “the nation,” “sovereignty,” “progress,” “colony,” “the West,” and “civilization.” I suggest here a dialectical notion in which indigeneity appears as an antithesis but, as such, it is always already a part of what it opposes. We can find out what indigeneity is not only by analyzing its relations to its others, but also by thinking of how indigeneity is contained in and comes into existence in and through its other—and vice versa. Through dialectical processes, I argue, indigeneity develops within the frame of the political economy. Moreover, indigeneity itself is dialectical in its relation to the political-economic frame: It contains, I argue in the last part, the contradictions of affirming and opposing the capitalist framework.

Drawing on an approach of political economy, one might ask if “paradox” is the right term here and whether the notion of “contradiction” would not better grasp the dialectical movements I am about to analyze. It is true that the idea of indigenous contradictions is what I want to outline in the following. However, “paradox” can be understood as a special case of contradictions, namely as a contradiction that is a necessary condition for something to come into existence, in this case indigeneity. That means that indigeneity is not only contradictive but that the contradictions of indigeneity are necessary features of it. For this reason, I use the notion of indigenous paradoxes. The insight that indigeneity consists of contradictions (and by extension, that it is paradoxical in nature) is not new. Scholars such as Ronald Niezen (2003) have noticed the contradictive nature of indigeneity, but in academic practice, the fact that indigeneity consists of contradictions has often been downplayed in order to avoid harming indigenous movements. However, as Luisa Steur (2005) has outlined, it is important to criticize the liberal culturalism that is well aware of these contradictions but does not openly address them. In order to widen the possibilities for solidarity and resistance, it is necessary to focus on “contradictions of indigenism and making them the starting point of critical analysis” (Steur 2005: 175).

Indigeneity is, as with other identities, always a matter of becoming and being but is never simply invented. Tania Li (2000: 151–153) has outlined in regard to Stuart Hall that indigeneity is a form of positioning. It is not something natural or inevitable, nor something invented or adopted in the sense that people are aware of its artificial character. As positioning, indigeneity “draws upon historically sedimented practices, landscapes, and repertoires of meaning, and emerges through particular patterns of engagement and struggle” (Li 2000: 151). I agree with this account and develop it further by arguing in this article that this positioning develops dialectically: the meaning of indigeneity is indeed historically sedimented, but it is more than a mere accumulation of meaning through history. Rather, I suggest that the notion of indigeneity developed as historical practices and meanings fostered and articulated contradictions, and sublated older meanings. Additionally, indigeneity came into existence as a category of struggle against and in engagement with the state, and it depends on both.

This contribution outlines this for the context of Indonesia, a country which was not a settler colony but nonetheless accommodates the indigenous paradox to some extent. However, I argue that there are more dimensions beyond the juridical and political process of the indigenous paradox, and I am going to outline two of them here. One is the narrative dimension of indigenous communities and activists through which indigeneity is made meaningful for local communities. However, the most important dimension of the indigenous paradox is its economic dimension, in which the paradox of being in and outside of capitalist relations emerges.

In order to relate the different dimensions of the indigenous paradox to each other, it is useful to draw on Louis Althusser’s notion of overdetermination (Althusser 2005: 87–128), a concept he borrowed from Freudian psychoanalysis. The basic idea of overdetermination is that a single event or observed effect is determined not only by a single cause but by multiple causes. Althusser argued that the superstructure has a relative autonomy from the base of economic contradictions. In contrast to orthodox Marxist accounts, Althusser argued that it is not possible to explain a historic event merely in regard to the base, that is, the economic contradictions. Rather, “the economy is the determinant, but in the last instance (…). From the first moment to the last, the lonely hour of the last instance never comes” (Althusser 2005: 112–113). This is also true for the case of indigeneity in the Indonesian context (and elsewhere): the superstructure, which also includes some dimensions of the indigenous paradox, namely the narrative, political, juridical, and (as we will see in the example of the Duri communities) the religious, for instance, is relatively autonomous from economic contradictions. But at the end of the day, the indigenous struggle is an economic struggle as it is concerned with land rights, that is, with the means of production, although indigeneity as an ideology does not portray itself as concerned with economic issues in the first place, but rather with cultural issues. For Althusser, contradictions are overdetermined as well. He argues with regard to Marx that economic contradictions never occur in their pure form but are shaped by history and current social, political and cultural circumstances, which give them their distinctive features.

This means that indigeneity expresses this overdetermination of economic contradictions as it is not simply an effect of the last determining instance, namely the economy. Rather, it translates economic contradictions into a language of culture and sovereignty claims. In other words, indigeneity is not class identity, nor is it independent from class identity. The relation between indigeneity and class has been the subject of debates in anthropology, for instance between Mahmood Mamdani (2000) and Bridget O’Laughlin (2000). Mamdani (2000: 44) has argued that in order to understand the dynamics of the colonial world and the production of the category of the native (and thus the indigenous), neither the political can be deduced from the economic nor the economic from the political. It is crucial to grasp the contradiction between the economic and the political. I rely on this approach for further analysis. However, I argue that this is only thinkable in a Marxist analysis if one assumes the relative autonomy of the superstructure (the political) from the base (the economic). Indeed, indigeneity is not a class identity as it cannot express economic contradictions in their pure form (if they ever appear in such a form). The relations of production are nonetheless addressed, as I will argue.

The overdetermination of the indigenous paradox is rooted, as I will argue, in the overdetermination of indigeneity as an identity through which economic contradictions assert themselves in other (usually cultural) forms. This line of thought provides a material-dialectical foundation for the indigenous paradox and, as I will outline, this holds true not only in settings where indigenous communities engage in violent conflicts over their land and rights. Drawing on the case of Duri communities in the uplands of South Sulawesi, I shed light on a case in which recognition of indigenous rights went relatively smoothly, at least when considering the local dimension of this process. However, here as well, the economy is the driving force, but other dimensions of the indigenous paradox are also crucial when explaining the emergence of indigeneity in the Duri highlands.

By taking political economy into account as the last determining instance, I aim to contribute to a critical investigation of indigeneity as a hyper-politics machine that offers symbolic-political solutions to political-economic problems (Goodale 2016: 442). While I agree with Mark Goodale’s critique here, investigating indigeneity and indigenous paradoxes as overdetermined in the Althusserian sense also allows for the progressive potential of indigeneity to be recognized, as economic contradictions and class struggles can be accommodated within indigeneity. In the dimensions of the indigenous paradox I am about to outline in this article, the overdetermination of the economic contradictions emerges. Althusser argues that the basic “contradiction is never simple, but always specified by historically concrete forms and circumstances in which it is exercised. It is specified by the forms of the superstructure (the state, the dominant ideology, religion, politically organized movements, and so on (…)” (Althusser 2005: 106). In other words, the indigenous paradox sheds light on the overdetermined contradiction between labor and capital, and in the case of indigenous movements, this especially addresses the question of land as a means of production, although indigenous discourses usually apply specific cultural attributes to the land, which are also important when one assumes that the superstructure is largely specific and relatively autonomous (cf. Althusser 2005: 113). In other words, indigenous ideologies and indigenous struggles are not reducible to mere phenomena of economic contradiction, but economic contradictions cause them in the last instance. As Nancy Fraser (1995) has argued, justice requires both recognition and redistribution. The indigenous struggle is about both, but by denying the economy as the last instance, it favors the first over the latter. A critique of indigeneity and indigenous movements from a dialectical-materialist perspective should shed light on this point but should also acknowledge the relative autonomy of indigeneity as a part of the superstructure.

## Indigeneity in the Indonesian context

Indigeneity developed into a political force in Indonesia in the 1990s when activists concerned with environmental issues and the dispossession of rural communities adopted the concept of indigeneity from transnational discourses. It drew, however, on local contexts and histories. Before shedding light on the case of Duri communities in South Sulawesi, I outline some developments and features of indigeneity in Indonesia and explain how they developed in history and the changing political economy of rural Indonesia.

In Indonesia, activists in the 1990s decided to translate the term “indigenous peoples” and “indigenous communities” into *masyarakat adat*, literally meaning “society of custom.” Custom here signifies systems of values, economy, culture, land management, and so on. *Adat*, in the widest sense of the term, means a cosmological order which gives sense to all everyday practices and institutions (Acciaioli 1985: 182; Moniaga 2007: 291–282). However, the conceptualization of *adat* has its roots in the late colonial order when Dutch scholars conceptualized *adat* as law (Burns 2004, 2007). *Adat* as a category of autochthon law in the Netherlands East Indies came into existence when Cornelis van Vollenhoven and scholars of the Leiden School collected data from missionaries and colonial officials, arguing that the colony was a space of distinct law with some crucial features. Most importantly, he argued that *adat* communities all over the archipelago had concepts of *hak ulayat* (right of avail). The *hak ulayat* is the right to communal property over which, in van Vollenhoven’s account, the *adat* law community should maintain and exercise its original social and political control (von Benda-Beckmann 2019: 404–405). On the other hand, scholars from the Utrecht School argued for an integration of autochthon communities into the market and rejected the idea of legal pluralism in the colony and the recognition of *adat*. As Tania Li (2010: 393–394) has summarized, the debate between the conservative Leiden School and the liberal Utrecht School was about the (economic) question of whether natives should become market subjects of colonial capitalism or not. The seemingly cultural arguments of the Leiden School therefore had economic implications. They feared that natives would be dispossessed of their land (as they might sell it), and thus, social unrest and upheaval would occur which might destabilize the colonial order. We can see here how indigeneity was not just the opposition or the other of the colonial state but also an integral part of it. It was conceptualized in regard to economic contradictions as van Vollenhoven saw that an incorporation of *adat* communities into the liberal market would lead to economic marginalization and potentially to social unrest.

In 1870, all land not under ownership under the civil code became the domain of the state. That meant that *hak ulayat* and other *adat* rights were largely ignored. Only if *adat* land were in “constant use” (*gedurig gebruik*), it would be regarded as non-free state land. This land was usually not sold or rented to others by the state, but indigenous ownership was also not recognized (Fitzpatrick 2007: 133–134). However, the colonial state recognized *adat* legal systems for *inlanders* and in directly ruled areas. Some Indonesian students and later members of the national movement such as Supomo, Maramis, and Subardjo were influenced by the conservative and integralist ideology of the Leiden school. They assumed that there was a single, basic “Ur-adat” that constituted a legal realm in the Netherlands East Indies fundamentally different to Western individualist and capitalist societies and their laws. This concept was crucial for the independence movement, but not all relied on reactionary concepts in which *adat* meant a local village system where people know their place in the social hierarchy and obey, and wherein the state should be based upon a similar concept. Sukarno, for instance, used the notion of *adat* and village life as well, but rather emphasized mutual cooperation, solidarity, and social equality (Bourchier 2007: 114–117). In other words, there was a conservative and a populist indigenism among those who struggled for Indonesian independence, and both deployed the notion of *adat* for their purposes (Bourchier 1998: 204–205, 209).

In independent Indonesia, the government sought an “Indonesian” foundation of land laws. *Adat* became the basis of the Basic Agrarian Law of 1960, but two important restrictions were made: the continued existence of *hak ulayat* had to be proven and state interests could always nullify *adat* claims. This development was due in part to leftist forces, especially from the Communist Party, which aimed at a general redistribution of land in Indonesia but paid little attention to *adat* communities as they prioritized the peasantry as a political force (Bedner and Arizona 2019: 419). When Suharto and right-wing fractions of the armed forces seized power, the political economy of Indonesia changed radically. An authoritarian developmentalist state was established and leftist organizations were outlawed. The new Suharto government sought to attract foreign capital for its developmentalist agenda. In order to do so, a Basic Forestry Law was issued in 1967. The law ensured the state’s control over all forests that were not privately owned and it even lacked the symbolic language of recognition of *adat* as found in the 1960 Basic Agrarian Law. *Adat* rights could not be converted into property rights. The state had the power to define land as forests and as the domain of the state, and this concerned about 70% of Indonesia’s land territory (Bedner and van Huis 2008: 181–183). From the 1980s in particular, the Suharto regime dispossessed numerous autochthon communities by declaring their land state forest and renting it out to companies. This process was said to be conducted in the interest of the nation and development. The communities in question were often labeled as “estranged” and “isolated” communities (*masyarakat terpencil*) both by the government in its development programs and in public discourse. State institutions defined “isolated communities” as nomadic, having poor housing and transportation facilities, engaging in swidden agriculture and other “unsustainable” resource use, with a lack of education and strong commitment to traditional beliefs (Li 2000: 154).

It was the political economy of original accumulation—a process in which the producers, namely the rural communities, had been separated from their means of production (land)—and, simultaneously, the discursive delegitimation of traditional ways of life that provided the grounds for resistance under the banner of indigeneity from the 1980s onwards. All over the archipelago, rural communities and activists organized under indigenous identities, as *orang asli* (“original people”), tribes, *masyarakat adat*, or as ethnic groups which had the stigma of being backwards and primitive (for instance: Robinson 1986; Robinson 2019: 473–475; Li 2000: 163–169; Moniaga 2007: 281–282; Maunati 2012; Tanasaldy 2012: 283–284). The reform era (*reformasi*), which began in 1998, saw a revival of tradition in Indonesian politics (Davidson and Henley 2007). On the one hand, local elites used *adat* and tradition to improve or maintain their political influence in local politics (i.e. Klinken 2007; Tyson 2010: 81–100; Thufail 2013; Klenke 2013). On the other hand, indigeneity became the foundation of a movement of the grassroots. As leftist approaches based on communism and socialism were de facto impossible since anti-communism had become a *raison d’état* since 1965 and Marxism was still outlawed in post-Suharto Indonesia, indigeneity became a promising alternative, also because it was well established in international discourses. In 1999, indigenous activists from all over the archipelago gathered in Jakarta for the first congress of the Indigenous Alliance of the Archipelago (*Aliansi Masyarakat Adat Nusantara*, AMAN). At that founding congress, the activists announced their famous slogan: if the state will not recognize us, we will not recognize the state (*Jika Negara Tidak Mengakui Kami, maka Kamipun Tidak akan Mengakui Negara)* (AMAN: undated)*.* At first sight, this indicates an oppositional strategy, a strategy of indigeneity against the state. However, from the very beginning of the indigenous movement, the relationship between the state and indigenous peoples was more complex. In the provocative statement announced by the activists in 1999, a dialectical relationship between indigenous peoples and the state was already outlined in a nutshell. As the statement was formulated negatively, *masyarakat adat* came into existence though the possibility of a negation of the state. Through such an identity, the “we” (*kami*) became a subject as it declared its ability to withhold recognition of the state. Consequently, the state appeared in AMAN’s view as a condition of the very existence of *masyarakat adat*, and the two entities of *masyarakat adat* and the state (*negara*) emerged as simultaneously constitutive to each other and as their respective negations (Tamma and Duile 2020: 276–277).

It is no surprise therefore that indigenous activists engaged with the state in order to achieve indigenous recognition. *Pengakuan* (recognition) of their autonomy and rights became the leitmotif of the movement. It became clear within the first years of the movement that indigenous communities in Indonesia did not seek sovereignty against the nation-state but rather autonomy (*otonomi*) within the state, which would allow the communities to organize on internal issues such as indigenous local economies. However, their notion of *otonomi* is partly contradictive as they recognize the state and its monopoly of violence but also demand the right to reject economic penetration from the outside and require the recognition of *adat* law and initiations for their internal issues, which would come at the expense of state laws and institutions (Acciaioli 2007: 295–307). Indigenous activists engaged with the state in order to achieve recognition. Most importantly, AMAN issued a lawsuit at the Indonesian Constitutional Court in order to review the status of *adat* forests, which fell within the state’s domain. In 2013, the Court ruled in favor of AMAN and decided that *adat* forest is not state forest. The state had to recognize *adat* land ownership, and this is also the case with land not in permanent cultivation on which *adat* communities claim their *hak ulayat* (Bedner and Arizona 2019: 423–424; Tamma and Duile 2020: 279). It is not surprising that indigenous activists intensified their relations with the state and its institutions.

During the 2014 election campaign, AMAN supported Joko Widodo (Jokowi), who was then elected as the new president. Jokowi promised to support indigenous communities in their struggle for land rights. Indigenous activists had therefore intensified their relations with state institutions as they saw the Jokowi presidency as a new opportunity for their struggle. Not only did indigenous activists engage in the Jokowi election campaign, they also developed strategic interactions with state officials (see on this issue more in detail: Affif and Rachman 2019: 461–466). In some cases, state officials were quite supportive. Siti Nurbaya Bakar, who became the minister for environment and forestry, for instance, cooperated with AMAN activists. Her ministry is important in the process of recognition because it is responsible for designating forest as *adat* forest. Another important ministry is the Ministry for Agrarian Affairs and Spatial Planning. However, relations with Sofyan Djalil, the minister of agrarian affairs and spatial planning and AMAN were difficult. Djalil stuck to the old paradigm that development in rural areas is most efficiently realized by large corporations and individual land ownership. He put an emphasis on the productiveness of land and declared that land held by the people but not used efficiently should be taken by the state. In his view, corporations with access to capital and knowledge are the better partners for the government (Maulana 2019). Meanwhile, Siti Nurbaya Bakar and president Jokowi emphasized the potential of local communities as entrepreneurs who could boost local economies. This image was also applied by AMAN, for instance by the former AMAN chair Abdon Nababan who argued for people’s agroforest areas (Nababan 2015).

However, land redistribution happened slowly and many indigenous activists became disappointed with the Jokowi administration. On 30 December 2016, 13,000 hectares were handed to nine indigenous communities in a ceremony in the state palace (Affif and Rachman 2019: 454). Some more communities followed in 2018, but Jokowi’s promise in the 2014 election campaign that by 2019 12.7 million hectares would be redistributed to indigenous communities was not fulfilled by no means at all (Tamma and Duile 2020: 280). Indigenous activists had to deal not only with the slow process of land allocation but also with a task that shifted their struggle to the level of the regencies (*kabupaten*). In 2016, in order to gain land titles from a natural reserve, AMAN activists agreed with a proposal from the Ministry of the Environment and Forestry that indigenous communities needed to be recognized first by their respective regencies (Affif and Rachman 2019: 463).

The current mechanism of recognition emphasizes the local level as there is no recognition of land titles before the communities in question are recognized by their respective regencies. In order to push local recognition, indigenous activists lobby on the provincial scale, hoping that provincial legislation or guidelines will accelerate legislation and recognition processes on the regency level. This emphasis on the local level is common in Indonesia’s decentralized political system, which started to emerge in 1999 after the downfall of the Suharto government and which especially stresses the importance of the regency level (Holzappel 2009). Decentralization has had tremendous impacts on indigeneity, both in terms of a revivalism of traditional elites and progressive indigenous struggles (for instance, de Jong 2009; Prabowo 2009). The forest sector was decentralized as well, with mixed results, and was eventually recentralized (Bullinger and Haug 2012). Therefore, indigenous activists now have to maneuver between different scales in order to achieve the recognition of their customary forests.

To summarize the history of *adat* and indigeneity in Indonesia, we can conclude that indigeneity as *adat* emerged as a concept in the colonial context when economic issues were addressed with cultural approaches. In independent Indonesia, *adat* became a foundation of genuine Indonesian identity, but was subordinated under the state and even completely ignored in the so-called New Order era. The New Order state dispossessed rural communities not only through original accumulation, when it took their land and rented it out to companies, but also in terms of ideology since political organizations under the banner of communism or socialism were outlawed. Indigeneity as a new emerging political identity became a means to express economic contradictions and to fight dispossession. However, it did and does so in terms of culture. The indigenous movement in Indonesia first adopted an oppositional stance towards the state, as it declared that it would not recognize the state if the state did not recognize indigenous communities. This recognition, however, also meant the acknowledgement of land titles—for many people the foremost issue. Therefore, indigenous communities and activists had to engage with the very state that they had threatened to refuse to recognize. The indigenous paradox also unfolds in the Indonesian context, but in comparison to settler colonies, indigenous activists could always claim to be an integral part of the nation. Their constitutive outside is rather the New Order state and its remnants in post-authoritarian Indonesia. This paradoxical notion of being, on the one hand, part of the nation and engaging with state institutions while on the other hand simultaneously denying the legitimacy of the state when it declares indigenous land as state forest is not only a matter of law and politics but, most of all, of the economy. At stake here is land as the most important means of production for rural economies. Land ownership is thus claimed against the state as the state’s claims of *adat* territory are denied, but also with the state as indigenous activists enter the state and seek to appear in its law. In terms of economic concepts, a notion of indigenous entrepreneurship represents an ideological conjuncture between parts of the political elite and activists, and it is through this conjuncture that institutional engagement becomes possible.

## Enrekang regency and the Duri: an overview

As this contribution has now shed some light on the national context of indigeneity in Indonesia, in the following, it aims to investigate the case of indigenous Duri communities. Many contributions have investigated how indigeneity and notions of “being traditional” have been deployed in conflicts over land and resources in Indonesia (e.g. Li 2000; Steinebach 2013; Bräuchler 2018; Robinson 2019; Fisher and van der Muur 2019). Certainly, these contributions have helped greatly to explain the phenomenon of indigeneity in Indonesia. However, many communities that now self-identify as indigenous do not find themselves in such highly conflictual settings. Indigeneity emerges through conflicts with the state and companies making land claims, but also through cooperation with these outside forces. In the case of the recognition of Duri communities as indigenous, conflicts are less prominent, but I argue that these cases can also help us to understand how indigeneity comes into existence and how the paradoxical dimension of indigeneity unfolds.

South Sulawesi was one of the birthplaces of Indonesia’s indigenous movement. In 1993, environmental activists and indigenous peoples met in Toraja and founded the Indigenous Peoples Rights and Advocacy Network, from which AMAN later emerged (Moniaga 2007: 281). The Toraja, with their iconic *tongkonan* and indigenous rituals, are one of the main ethnic groups in South Sulawesi. They live in the uplands and are mostly of Christian faith. The other dominant groups are Bugis and Makassarese, both Muslim groups from the lowland. While there are numerous ethnographic accounts focused on these major groups (e.g. Nooy-Palm 1986; Rössler 1990; Hollan and Wellenkamp 1996; Pelras 1996), smaller groups are far less frequently considered in ethnographic research on South Sulawesi. Some of these lesser groups live between the major groups, between the lowland and upland, for instance in the regency of Enrekang. In local languages, this area is often referred to as *Massenrempulu* which was also the name of a federation of petty kingdoms and translates literally as “at the edges of the mountain.” Besides the Enrekang ethnic groups in the central part of the regency, there are two more groups, namely the Maiwa in the southern, lowland part, and the Duri which dwell the northern upland up to the borders of Toraja. All groups are of Muslim faith which distinguishes them from the Toraja, but in terms of language especially the Duri share similarities with the Toraja (Batong 2007; Hadrayani and Karim 2019; Tamma and Duile 2020: 281).

Enrekang was among the first regencies to pass a local regulation (*peraturan daerah*, *perda*) for the recognition of indigenous communities. The *perda* was issued in early 2016. A number of things made this legislation possible. In Enrekang, indigeneity had not previously been appropriated by traditional elites (like, for instance, in nearby Toraja) who might compete with AMAN on the issue of indigeneity. Moreover, indigenous activists had good ties with local politicians. Also, there were no latent conflicts between the state and companies on the one hand and indigenous communities on the other, despite the fact that local communities frequently violated laws and entered state forests. Finally, the common goal of economic extraction and growth provided an ideological consensus between the activists, local communities and the local state apparatus.

In the following, the article sheds light on the political process of recognition and how indigeneity emerged in the Duri highlands. Thus, other dimensions of the indigenous paradox are outlined, especially the economical stance of being part of the capitalist economy and seeking autonomy for an alternative indigenous economy.

## The politically and juridically paradoxical formation of indigeneity in Duri communities

In Indonesia, Indigeneity is sometimes at odds with Islam as some activists perceive Islam as a modernizing force that destroys traditional worldviews. This is in accordance with some Western views that emphasize that indigenous peoples are only fully indigenous when they maintain their traditional worldviews against modern religions. While generally careful when it comes to the sensitive topic of religion, some indigenous activists expressed their support when *kepercayaan* (an umbrella term for traditional beliefs) was recognized as an official belief in Indonesia in 2017. Occasionally, indigenous activists complained about aggressive efforts to convert animists into Muslims, for instance among the Badui in West Java. However, many indigenous communities in Indonesia are of Muslim faith and often Islam has even shaped their indigeneity in a profound way, as I will outline in regard to the Duri communities: Islam was indeed a modernizing force with a tremendous impact in the Duri highlands. Political and juridical contradictions in the process of recognition are often linked to the issue of Islam, and on the one hand, indigenous activists have found ways to incorporate Islam into their agenda, while on the other hand, Islam has profoundly shaped what is now called indigeneity among the Duri.

As elsewhere in Indonesia, it is not ethnic groups who are recognized as indigenous but communities. These communities consist of one or a few villages and are usually much smaller that ethnic groups. These communities can, after they gain acknowledgement from their regency’s government, apply for *adat* forest land titles at the Ministry of Agrarian Affairs and Spatial Planning. The large network of politicians and religious authorities that indigenous activists in Enrekang are embedded into is probably a main factor in why Enrekang was among the first regencies to adopt a local regulation (*peraturan daerah* or *perda*) on the acknowledgement of indigenous communities. In particular, Pak Budi, the head of AMAN Enrekang, is crucial in this regard. Being a member of PAN (*Partai Amanat Nasional*, National Mandate Party), a political party affiliated with modernist Islam, he has strong ties to both politicians and Islamic clergy. This was particularly important in 2015 when conservative Muslims in Enrekang feared that the acknowledgement of indigenous communities would come at the expense of “proper” Islam, as indigenous communities would revitalize traditional beliefs (known as *Aluk Tojolo* in the Duri upland). Countering these accusations, indigenous activists always stressed their Islamic beliefs. In one conversation with Pak Budi, he stressed the importance of Islam against everything that is irrational and emphasized what he acknowledged as the foundations of AMAN and the indigenous struggle: Islam, the Indonesian Constitution (*Undang-Undang Dasar*), and the national ideology of *Pancasila*. Also, *adat* heads in several Duri villages stressed in our conversations that traditional rituals are always embedded into Islamic contexts: It is always Allah who is addressed in the indigenous rituals and often *adat* experts and Islamic clergy perform rituals together.

Pak Budi explained that during the Darul Islam rebellion in the 1950s and 1960s, traditional beliefs were outlawed and any ritual relation with spirits was subject to punishment. However, for indigeneity as Pak Budi understands it, traditional beliefs are unimportant. Indigenous activists in Enrekang in general are eager to portray indigenous communities as a part of the local community and the Indonesian nation, and this is also made clear in the *peraturan daerah*. At the beginning of the document, indigenous communities (in the document as “customary law societies”, *masyarakat hukum adat*) are said to be Indonesian citizens with “special characteristics,” probably in cultural terms. A further criterion is that they live in “harmony according to their customs” and have “strong relations to the land and environment.” Here it is already clear that the *peraturan daerah* refers to transnational discourses on indigeneity in which indigenous peoples’ relationship to the land and their cultural distinctiveness is stressed, and to Indonesian nationalism and the Indonesian nation as the very framework of their identity. Indigenous identity in Enrekang is evoked in accordance with the nation and thus points toward spaces of indigenous distinctiveness within (and not against) the nation. This fits well into the concept of the citizenry of a plural state (*negara majemuk*) that indigenous activists have stressed from the very beginning (Acciaioli 2007: 305). Also, indigenous rights to natural resources as communal or individual rights which “originate from their origin” and stem from their “distinctive social systems and cultures” are mentioned in the *peraturan daerah*.

In order to gain acknowledgement, the *peraturan daerah* outlines a process for communities in which they are identified by a committee investigating whether the community in question has a history of being an indigenous community with customary territory, customary law, “treasures” or cultural artifacts, and indigenous institutions. According to the documents of the recognition of the Marena and Orong, two Duri communities who gained acknowledgement in 2018, and the data collected by indigenous activists for the committee, indigenous history is narrated as a genealogy of the *adat* heads, but is also embedded in hegemonic narratives of the nation. It is, for instance, written that *adat* heads of the Orong had engaged in the war against the Dutch, even suggesting that they were close to the (Indonesian) government. Also, when the data mentions indigenous rituals, these rituals are not only embedded in Islamic contexts but are also portrayed as a sign of being grateful to the “almighty God,” using the phrase *Tuhan Yang Maha Esa* which refers to the national ideology of *Pancasila* which itself uses the phrase *Ketuhanan yang Maha Esa* as a concept of a divine entity.

All this demonstrates that indigenous sovereignty is only thinkable within the sovereignty of the Indonesian nation and its narratives. The local regulation as well as documents from the committee and indigenous activists who gathered data for the committee suggests that indigenous communities are integral parts of the nation, but it is just this constellation that enables them to gain control over their land. With this strategy (and it is not merely a strategy as the AMAN activists in Enrekang genuinely believe in it), it was not only possible to gain support for the *peraturan dearah* from all factions within the regency’s legislature and to appease pious Muslims, but also to get the recognition of six indigenous communities in 2018 and three more in 2019. In total, 20 communities in Enrekang have become AMAN members. AMAN is heavily engaged in the procedure of recognition. As AMAN activists provide the data on the communities seeking recognition, being an AMAN member is a necessary part of seeking recognition from the regency.

AMAN has its own definition of indigenous communities, and in the context of South Sulawesi, indigeneity is a contested concept. As traditional elites engaging in local politics are eager to deploy the concept of indigeneity, AMAN activists distinguish between indigenous communities on the one hand and sultanates and kingdoms on the other hand. As Rukka Sombolinggi, the current head of AMAN, declared at the AMAN council of South Sulawesi in 2019, for instance, sultanates and kingdoms are not *masyarakat adat* as they have cooperated with colonizers and have also exploited indigenous communities. Indigenous communities are those who have always been marginalized, those “who have always lost in history”, from colonialism to the New Order, and are now about to struggle for their rights. In the Duri uplands, there had also been some petty kingdoms with local hegemony prior to the Darul Islam rebellion. Though some of the successors of the petty sultanates tried to register as AMAN members, their memberships were denied because of their history as kingdoms.

AMAN promotes the image of rather egalitarian, marginal communities, but when Duri communities fit into this image, their current state is, contrary to the indigenous claim, not an authentic, constant feature. It is rather the result of the troubled history of the Duri highlands. It is difficult to draw a clear line between kingdoms and indigenous communities, and social stratification was a crucial feature of all of these groups. It was not before 1906 that the Dutch established control in the Duri area and they kept most of the traditional hierarchy intact and cooperated with adat heads, outlawing debt bondage and other forms of slavery. The Dutch introduced a clear distinction in South Sulawesi between nobles and all others (Pelras 1996: 276). However, probably the most radical change in Duri societies came in the 1950s and early 1960s during the Darul Islam rebellion. This rebellion, which is often depicted as a serious threat to the Indonesian nation in state narratives, had a stronghold in the Duri area: the rugged terrain was ideal for guerilla warfare and the troops received much support from the Duri. The Darul Islam movement aimed for an Islamic socialism including moderate land reform. Social inequality was rejected and nobles lost their *adat* positions (Pelras 1996: 284). Debt bondage and slavery were abolished among Muslims.

The abolishment of all hierarchies accompanied the war, and the Duri often found themselves the victims. As many ordinary Duri peasants supported Darul Islam because of their egalitarian approach, the Indonesian Army set villages on fire when the inhabitants cooperated with Darul Islam. In South Sulawesi, the Indonesian Army was depicted as an alien, Javanese force which only had the support of aristocrats and nobles (van Dijk 1981: 155). Contrary to official Indonesian narratives, many interlocutors (both indigenous activists and ordinary villagers) still had good opinions of the Darul Islam rebellion, stressing its egalitarianism. Indigenous activists never denounced the Darul Islam movement. After the defeat of Darul Islam, local nobles tried to re-establish their positions. However, as modernist Islam became influential, this time through Muhammadiyah, traditional *adat* hierarchies were still in a difficult position. The outcome was rather egalitarian communities (especially when compared with the socially stratified communities in nearby Toraja), except for hierarchies between male and female. On the downside, some *adat* institutions disappeared and some communities could therefore not apply for indigenous community status. However, in those communities with intact *adat* institutions, those institutions had lost some of their power and as a result they fit much better into AMAN’s image of egalitarian indigenous communities: ironically, it was the modernizing, anti-traditional Darul Islam who shaped Duri societies in this way.

This helps us to outline some dimensions of the indigenous paradox in Enrekang and the Duri uplands. In political terms, the process of recognizing indigeneity acknowledges the state and the nation in the first place. Contrary to AMAN’s famous statement that they would not acknowledge the state if the state did not acknowledge indigenous peoples, AMAN activists in Enrekang engaged in the political framework of local state institutions as the given and acknowledged framework, lobbying for the *peraturan daerah* and thus for the recognition of its member communities. In the *perda* and the documents of the recognition process, indigenous communities emerge as a constant part of the nation, but as distinct in cultural manners. These cultural features constitute rights, and this is where the sovereignty of indigenous communities comes in. This sovereignty means, above all, land rights, as *adat* forest, should be in the hands of indigenous communities. The whole process of recognition revolves around the issue of the land rights of the communities in question. Here, the indigenous paradox unfolds as follows: only through the prior recognition of the state and the nation is it possible to gain land from the state and thus establish indigenous autonomy.

In terms of history, another paradoxical dimension comes into focus: indigeneity is narrated as an identity of a marginal and egalitarian social group which distinguishes itself from hegemonic parts of society. In Enrekang, indigenous communities also have to be similar to other parts of the population, most of all in terms of religion. Being Islamic is crucial for their recognition, whereas difference is evoked through other cultural characteristics such as *adat* institutions and material culture. Only when similar in terms of religion can the communities occupy a space of legitimate and acceptable difference. Also, the idea of egalitarianism is contradictory to the claim of being traditional, as egalitarianism is a rather new development (which took the Duri societies from a society with slaves to an egalitarian Islamic socialist society in only five decades). However, indigenous activists and people in the recognized villages subscribe to narratives which do not oppose indigeneity and Darul Islam rebellion (as they do not oppose Islam in general). AMAN activists act on behalf of both tradition and egalitarianism, and this is clearly a paradox, but just as with the paradoxical formation of their relation to state and sovereignty, this paradox is crucial for their agency. And it is the affirmation of egalitarianism which makes their version of indigenous traditionalism possible.

## The Paradox of Indigenous Economy

In the first part of this article, I argued with regard to Mark Goodale (2016) that indigeneity offers symbolic (cultural) solutions to political-economic problems. I now return to this issue and explain how this happened in the Duri highlands. It is indeed the case that indigeneity obscures economic processes, but on the other hand, it is this obscuring through the language of indigeneity that makes new forms of economic organization possible. Indigenous economies, as they are about to emerge in the new indigenous communities, point towards contradictions but also offer a means for a more egalitarian organization of local economic processes.

In the Duri highlands, manifest and violent conflicts over land and other resources are largely absent and have been so over recent decades. The *peraturan daerah* and the process of indigenous recognition were not carried out in order to settle a conflict between indigenous communities and companies as was the case in Bulukumba, for instance (Tyson 2010: 129–153; Fisher and van der Muur 2019). Rather, the conflict between local populations and the state was low key, as people were aware that the state had claimed control of their customary forests, but they nonetheless entered the forbidden areas in order to collect forest products. In some villages such as Uru or Marena, for instance, people planted fruit trees and even coffee in state forests, maintaining and harvesting their products with some caution. Occasionally, the Forestry Department would catch farmers in state forests, but overall, the state was largely absent. In conversations, indigenous activists portrayed the state much more as a threatening force than local people did, as the activists stressed their opposition especially to the New Order state as a constitutive other for their indigeneity. Some areas were dedicated to timber production, but the forests utilized by local communities were neglected areas. Underneath the process of indigenous recognition, an ideological conjuncture arose between local state institutions, indigenous activists, and people in the respective villages. Their common goal was what Tania Li (2007) called “the will to improve,” and it will be outlined in the following.

Just as Pak Budi was a major actor mediating between the local AMAN branch, his political party and thus other parties in the legislature, Pak Azis, an assistant to the regency head (*bupati*) was such a mediator from the other side. When talking about the process of recognition, he speaks of “stakeholders”: the regency government, village governments, activists, NGOs, and potential investors shared the goal of economic development. In his words, the customary forest should be managed in order to “push” the economic development of the indigenous community without destroying the environment and the forest. He said that the customary forests could become tourism sites. Also, the government supported the communities by buying 1,000 seeds each of durian and nutmeg trees to plant in the *adat* forests. Pak Azis wants the forest to be “really productive,” and the indigenous management reconciles production and protection for him. In his view, the whole process of recognition should “lead to a boost for the indigenous economy.” A similar motivation also prevailed in the recognized indigenous villages. The indigenous community of Orong, for instance, became an AMAN member in 2012. Activists back then referred to AMAN’s goals, especially their overall goals of becoming economically independent (*mandiri secara ekonomi*), sovereign in political matters (*berdaulat secara politik*), and culturally dignified (*bermartabat secara budaya*). What persuaded the villagers most was, according to the Orong’s *adat* head, AMAN’s slogan of becoming *mandiri secara economi*, and especially the idea of gaining recognized control over the *adat* forest. During the author’s stays in late 2019, people in Orong discussed planting coffee trees in their *adat* forests and establishing a tourism site. However, first they had to establish a road to the forest.

What is at stake here is *hutan adat*, that is, forest, but from the concepts delineated by activists and villagers, there is no clear-cut distinction between forest and agricultural land. It is true that the recognized *adat* forests should be maintained as forests, but this does not rule out the use of forests as agricultural forests where fruit trees or coffee trees are integrated into the forest to a quite large extent, or the use of a forest as a tourism site. In fact, indigenous forest management might serve as an excuse for agricultural activities in forest areas that previously have not been designated for agricultural production.

Indigenous activists have organized workshops with recognized communities from Enrekang and Toraja as well as with foreign NGOs in order to establish criteria for indigenous coffee labels. They hope that certified indigenous production with traditional organic fertilizers can add value to their coffee they want to sell to big cities and maybe even overseas. After the Orong forest was, as Pak Denny, the village head put it, “liberated” (*dibebaskan*) in 2018, not much changed in terms of forest use. It was common before the “liberation” to cultivate cloves, mangoes, and even pepper and dragon fruits in the forest, and people continued to do so as it was now legal. This raised some concerns about conservation, but as long as there was no road for cars to get to the forest (which is about a 40-min motorbike ride from the main village), exploitation remained limited. Development of infrastructure had been outlined as a necessary condition for successful exploitation of the forest. The Orong community also now benefits from a contract with a resin-extracting company which operates in parts of the *adat* forest where pine trees grow, but *adat* heads emphasized that the company now has to comply with adat rules, which forbids resin extraction from young trees.

However, despite all the plans and initial cooperation between the government, the company, and the communities, the idea of an indigenous economy often remains vague, encompassing different hopes and possibilities. AMAN has established indigenous economic groups (*kelompok ekonomi masyarakat adat*) in several Duri villages, which are meant to be in charge of the customary forest, its exploitation, and protection. These groups were established in 2019 and called *Pammesatan*, which means “unity” in Duri. By 2021, 40 families had joined the *kelompok ekonomi* in Orong and 32 in Marena. The aims of these groups were often a topic of conversation, and people referred to notions such as *mengingkat kapasitas* (capacity building), *hidup kesejahteraan* (living in wellbeing), and *pengembangan diri* (self-development). In other words, they adopted the language of entrepreneurship, and this language and these concepts express the ideological conjuncture with the local state apparatus. The *kelompok ekonomi* is equally open to all members of the *komunitas masyarakat adat* who wish to participate. This follows a narrative that all members have equal *hak ulayat* as long as they are members of the community. Therefore, mutual assistance (*gotong royong*) is, within the structure of the *kelompok ekonomi*, not a means of labor mobilization on the basis of political status, as has often been the case in Indonesian history (see on that issue: Bowen 1986: 548–549).

When asked why people joined the *kelompok ekonomi*, both activists and villagers said that they like the group’s foundations of *gotong royong* and *kebersamaan* (togetherness). It is important here to contextualize the notion of *gotong royong*. During the New Order, *gotong royong* served as a national construct to legitimize state interference into village life and the mobilization of rural labor (Bowen 1986: 555). This state interference was, however, quite weak in the Duri highlands. The term *gotong royong* was nonetheless known among the Duri. It was popularized again by AMAN activists as a means to describe indigenous features and village autonomy. In this regard, it is reminiscent of Mohammed Hatta’s notion of *gotong royong*, where mutual assistance is depicted as an original feature of village democracy against feudalism (Bowen 1986: 549–550).

To people in the Duri villages and indigenous activists, *gotong royong* and *kebersamaan* are indigenous values which are already strong in the community. However, if these values are already strong, the question of why they need the *kelompok ekonomi* arises. The culture of *gotong royong* is usually put into practice when a household needs help, but *gotong royong* did not have a permanent, organized form. In this regard, the *kelompok ekonomi* represented something new for the people in Orong and Marena. The institutionalization of *gotong royong* in the *kelompok ekonomi* came with the control of the *adat* forest as a crucial means of production that is owned and administered together. In practice, decisions over forest exploitation need the approval of the *adat* elders, but they usually approve what the group decides. The *adat* elders’ status is thus rather symbolic and does not allow for personal accumulation. Also, the groups had plans to buy other means of production, namely coffee roasting machines, facilities for drying the beans and harvesting from the trees. This is remarkable insofar as collective ownership of movable property beyond families and kinship groups is historically uncommon in Indonesia (Henley 2007: 101) and is also something new in Duri communities. It just appears as a part of *adat* because it is framed as a part of mutual help and assistance. Also, indigenous labeling as a means to add value to the products is in collective ownership. In other words, the *kelompok ekonomi*, even though their members adhere to and express entrepreneurial aims, offers the possibility of a new solidarity economy. This would be, however, embedded in wider capitalist networks, and the overall foundation of state capitalism has to be acknowledged first in order to gain room for indigenous alternative economies. This is where the economic dimension of the indigenous paradox becomes visible: the possibility of an indigenous solidarity economy in the Duri highlands can only emerge when recognizing the hegemonic capitalist and entrepreneurial approach of the state and its institutions. The indigenous economy cannot be outside capitalism as their goods are distributed through the markets, but they make possible new forms of local appropriation of nature.

## Conclusion: the economic indigenous paradox and its dialectics

This article suggests that Islam (in the form of religious party politics, historical forces, and Islamic ways of life in the indigenous villages), NGOs, regional autonomy and national discourses, and the concept *gotong royong* have all become sites of articulation of indigeneity and its contradictions. Moreover, indigeneity as it now appears in the Duri highlands inevitably consists of contradictions in itself; its contradictions are a condition for its existence. Therefore, I have called these contradictions “paradoxes.”

I have argued that paradoxes of indigeneity are a form of a dialectical process (rather than an issue of relation and difference). Indigeneity always appears as antithesis: First as *adat* as the cultural other within what it opposes (Dutch colonialism). This does not mean that *adat* is simply the opposite of colonialism. In fact, it was an integral part of the colonial endeavor as recognition of *adat* could serve to stabilize the colonial order. However, indigeneity was the negation of the colonial state insofar as *adat* was developed with regard to specific economic concerns that expressed economic contradictions within the colonial state (Li 2010). Eventually, indigeneity was sublated in Indonesian nationalism as the Indonesian nation was a synthesis of the Western concept of the nation and indigenism as its inner form, only to emerge again as a new antithesis (as *masyarakat adat*, “indigenous peoples”) to the state and its economic-political practices during the late New Order. In its engagement with the (post-) *reformasi* state apparatus, it sublated the notion of indigeneity as antithetical to the state since indigenous people now sought sovereignty within the state and became a part of the political economy that the state supports. In other words, indigeneity is a form of positioning depending on historically developed practices and meanings (Li 2000), but this evolved in a dialectical pattern.

As I have demonstrated in the part on indigeneity in the Duri highlands, indigeneity now reconciles with the state and its apparatus, but points toward new contradictions between affirming entrepreneurialism (which could fit into a neoliberal framework but does not necessarily have to) and the emergence of a local economy of solidarity. The co-production of entrepreneurial capitalism, the ideology of economic growth, and indigeneity in the Duri highlands have resulted in an economic indigenous paradox. As indigenous activists and people in the now indigenous villages begin to outline “indigenous” economies of solidarity (which are, contrary to their claims, not simply traditional ways of working together but are shaped by history and current political conditions), they can do so only by embracing the fundamental capitalist paradigms of the state apparatus. It is only through their common aims of market-oriented indigenous extractivism and capitalization of nature that the alternative spaces of local economies become possible because the common ideas and aims of boosting the rural economy laid the foundation of indigenous recognition. It is crucial, therefore, not only to understand that indigeneity is a political identity (rather than an economic one) but to grasp the contradiction between the economic and the political, or, to be more precise, between class identity on the one hand and native or indigenous identity on the other (see Mamdani 2000). Indigeneity is embedded into economic contradictions, and while not simply an economic identity, it is a product of it, and it relates to the political economy in contradictive ways. If indigeneity has a relative autonomy from the economic base, one can argue that it developed through economic processes but maintains and expresses contradictions. The relations between the political (or, to be mere precise, the cultural-political) identity of indigeneity and the economic-political identity of the peasantry is, I conclude, not just contradictory, it is also dialectical: indigeneity sublates peasantry in a certain sense; it abolishes it in the sense that indigeneity is an alternative political identity; and it maintains it in the sense that it provides a tool for economic struggles. The same holds true for indigeneity in the local setting in Enrekang. Indigeneity sublates, for instance, the Darul Islam movement. It opposes and rejects it because indigeneity rejects the aim of an Islamic state and supports the nation state, but it preserves the egalitarian notion of the Islamic movement. However, new contradictions can emerge within indigeneity.

What political-economic realities are thus obscured (but in some way also made tangible for ordinary people) in institutions of indigeneity? First, AMAN provides a narrative for local people’s separation from the means of production (land). The New Order declared land to be state forest in an act of heteronomy. The reason why local people should have direct control over the forest is, in the narratives of indigeneity, because they are indigenous, the original inhabitants, who had been there long before Indonesia’s establishment. In their view, indigenous communities constitute the state in its diversity, and therefore, the New Order violated the state’s obligation to indigenous communities. This narrative makes the original accumulation through the state apparatus tangible to people in the Duri villages, but it also obscures this economic process by rendering it a cultural-political rather than an economic-political issue. Secondly, there are now grassroots indigenous institutions such as the *kelompok ekonomi*. This institution clearly coordinates economic relations and, as I have argued above, set the condition for a rather egalitarian organization of economic relations. On the one hand, indigeneity obscures this economic process. Instead of consciously creating new economic forms of organization, people think that they are only “going back” to an original form of economy that existed before the state separated them from the *adat* forest. However, as I have argued with regard to Tania Li (2000), indigeneity is a positioning, drawing on the historical processes in the Duri highlands, and is, in its current form, actually a new identity. Collective ownership of the means of production is made tangible and desirable through the concept of indigeneity. In this sense, the symbolic solution for a political-economic problem is in itself already political-economic, albeit that it does not appear through a political-economic identity. This leads to economic contradictions that are a necessary condition for indigeneity. Indigeneity means equality, but it also means tradition. It means criticizing the state and its original accumulation, but it also means seeking recognition from the state and expressing itself in the language of the state, that is, economic growth and commodification of natural resources.

The paradoxical economic situation also emerges on the national scale. On the one hand, activists argue in terms of economic growth and stress their aim of rural development, which appears as compatible with the government’s notion of economic development (Nababan 2015). On the other hand, AMAN occasionally criticizes the political economy, for instance when advocating for land reform and engaging with other marginalized groups (AMAN 2020). This economic paradox is embedded in the political paradox of simultaneously acknowledging the state and claiming indigenous autonomy. In the Indonesian context, indigeneity is no longer constructed against the state, as indigenous activists have engaged with state institutions and acknowledgement from those state institutions has begun a process of recognition.

This article has contributed to research in the indigenous paradox (Bens 2020) and expanded the paradox beyond law, especially to the economy. The paradoxical situation of being part of, supporting and simultaneously claiming to be outside the capitalist realm, is an expression of economic contradictions translated into the language of indigeneity. It is this very language that provides the possibility for alternatives, and time will tell if these alternatives of indigenous economy will come into existence or if indigeneity will become just a tool for neoliberal entrepreneurship in which certain individuals might benefit from indigenous land and enterprises but no fundamental alternative emerges. The indigenous paradox points towards its own overdetermination. As a contradictory constellation, it speaks of culture while addressing economic questions and expresses itself in and through economic contradictions. In the Duri highland, this economic contradiction consists of the state’s grasp on land as state forest, while the prevailing ideology suggests the need to appropriate nature efficiently. As the state has failed to do so, indigenous entrepreneurship and vague ideas of a solidarity economy emerge as “indigenous” answers to the contradiction of state claims and the state’s economic agenda of economic growth. By introducing new ways of organizing the local economy, it points towards the basic contradiction between ownership and productive forces, and suggests sublating this contradiction by introducing a solidarity economy. However, this approach will only become truly emancipatory if the *kelompok ekonomi* as a form of solidarity economy will develop into a universal category for organizing the economy beyond the particular struggle of indigenous peoples.

What do the constituting contradictions of indigeneity mean for grassroots activism? Indigeneity as a political force can benefit from taking its own contradictions into account. Only when recognizing its paradoxical character can it convincingly denounce those who speak on behalf of indigeneity and simultaneously in the name of the (local) state, because the contradictive character of indigeneity can show how these traditional elites in the local state apparatus veil the constituting contradictions of indigeneity and rather act on behalf of their economic interests. The notion of indigeneity as social equality must acknowledge the contradictive history of indigenous communities, as indigeneity otherwise often means going back to (imagined) traditional hierarchies. Taking its own contradictions into account, recognizing and embracing them might be a helpful strategy for indigenous grassroots activism against essentialization and is a powerful tool against cooptation by traditional elites. Focusing on the contradictions of indigeneity can, as Steur (2005) has outlined, also widen the possibilities of solidarity and resistance: the indigenous movement, once aware of its own contradictions, might easier find some common ground with other rural movements and become more resistant to the threats of ethnic exclusivism.
